# Integrating sEMG and IMU Sensors in an e-Textile Smart Vest for Forward Posture Monitoring: First Steps

**DOI:** 10.3390/s24144717

**Published:** 2024-07-20

**Authors:** João Martins, Sara M. Cerqueira, André Whiteman Catarino, Alexandre Ferreira da Silva, Ana M. Rocha, Jorge Vale, Miguel Ângelo, Cristina P. Santos

**Affiliations:** 1 Center for Microelectromechanical Systems (CMEMS), University of Minho, 4800-058 Guimarães, Portugal; a88354@alunos.uminho.pt (J.M.); asilva@dei.uminho.pt (A.F.d.S.); 2 Center of Textile Science and Technology (2C2T), University of Minho, 4800-058 Guimarães, Portugal; whiteman@det.uminho.pt (A.W.C.); amrocha@det.uminho.pt (A.M.R.); 3 LABBELS-Associate Laboratory, University of Minho, 4800-058 Guimarães, Portugal; 4 Valérius-Têxteis, SA, Rua Industrial do Aldão, Apartado 219, Vila Frescaínha, S.Martinho, 4750-078 Barcelos, Portugal; jorge.vale@clothius.pt (J.V.); miguelangelo@valeriushub.com (M.Â.)

**Keywords:** e-Textiles, Wearables, Textile dry sEMG sensors, IMUs, Forward Head Posture

## Abstract

Currently, the market for wearable devices is expanding, with a growing trend towards the use of these devices for continuous-monitoring applications. Among these, real-time posture monitoring and assessment stands out as a crucial application given the rising prevalence of conditions like forward head posture (FHP). This paper proposes a wearable device that combines the acquisition of electromyographic signals from the cervical region with inertial data from inertial measurement units (IMUs) to assess the occurrence of FHP. To improve electronics integration and wearability, e-textiles are explored for the development of surface electrodes and conductive tracks that connect the different electronic modules. Tensile strength and abrasion tests of 22 samples consisting of textile electrodes and conductive tracks produced with three fiber types (two from Shieldex and one from Imbut) were conducted. Imbut’s Elitex fiber outperformed Shieldex’s fibers in both tests. The developed surface electromyography (sEMG) acquisition hardware and textile electrodes were also tested and benchmarked against an electromyography (EMG) gold standard in dynamic and isometric conditions, with results showing slightly better root mean square error (RMSE) values (for 4 × 2 textile electrodes (10.02%) in comparison to commercial Ag/AgCl electrodes (11.11%). The posture monitoring module was also validated in terms of joint angle estimation and presented an overall error of 4.77° for a controlled angular velocity of 40°/s as benchmarked against a UR10 robotic arm.

## 1. Introduction

With rapid technological advances, the development and utilization of wearable technologies have experienced exponential growth. This market has already proven its worth, with an estimated global value of USD 32.63 billion in 2019 and expected annual growth of 15.9% until 2027 [1]. Wearable technology offers a wide range of applications, such as tracking user movement using accelerometers and gyroscopes. Thus, these technologies can become valuable tools for the prevention of work-related musculoskeletal disorders, for which poor posture plays a crucial role.

The rise of new communication technologies like smartphones, tablets, and computers in workplaces has led to more frequent ergonomically incorrect postures, including FHP. FHP is linked to increased thoracic kyphosis (curvature of the upper spine) [2] and upper cervical vertebrae hyperextension [3,4]. This is a pressing issue, since in 2020, there were 203 million cases of neck pain globally, with an expected increase of around 32.5% by 2050 [5]. This condition imposes a significant economic burden. For example, in 2013, in the United States alone, costs related to neck pain reached USD 87.6 billion, with an annual increase in treatment costs of 6.5% [6].

To mitigate this postural behavior, monitoring methods are employed to assess and prevent postural risks. Assessment can be done through observation by a specialist after the worker completes the task. However, this method is subjective due to the specialist’s assessment, making it challenging to establish consistent patterns among subjects [7,8], and feedback is not provided in real-time. Instrument-based methods that use sensors on body segments provide more objective evaluations of posture by collecting data. Over the years, several studies have investigated the impact of FHP on the muscles of the cervical region through the acquisition of sEMG signals, concluding that this postural behavior is associated with increased muscle activity in this region. Changes in the curvature of the spine have also been assessed using inertial technology, with the results showing an increase in curvature, especially in the thoracic region of the spine. Overall, in terms of sEMG studies, Park et al. [9] observed increased activation of the upper trapezius during forward bending. Similarly, ref. [10] found higher muscle activation in the cervical region during FHP compared to corrected posture by monitoring the upper trapezius and cervical erector muscles. These conclusions are supported by the results of Malmström et al. [11], who observed elevated sEMG activity in the upper and lower trapezius and the serratus anterior muscles during slouched posture, as well as increased energy expenditure.

To evaluate the curvature of the spine, some systems have proposed the use of IMUs to quantify these changes. Wang et al. [12] proposed a system to monitor the existence of compensatory movements during arm raises. The proposed system features two IMUs, placed on the T1 and T5 vertebrae, to assess the curvature of the thoracic portion of the spine and the existence of FHP. In [13], the authors proposed a system that also uses IMUs to monitor head posture. The inertial units were placed on the left, right, and back of the neck, allowing determination of the orientation of the head in the frontal, sagittal, and transversal planes. Ref. [14] proposed a system containing two IMUs placed at the upper and lower part of the spine to monitor both the coronal and sagittal planes.

For a wearable device to be successful, ensuring user comfort, usability, and, particularly, practicality, is paramount. Notwithstanding, some of the presented systems still face some challenges regarding the integration of the electronics into the textile, potentially resulting in user discomfort and diminished device usability. One potential solution to improve these issues is using e-textiles, which consist of textiles coated with conductive materials that provide them with electrical properties. These types of fibers have been used in [15,16,17] to connect various electronic components and sensory modules in wearable devices. In these studies, the fibers were coated with metallic materials such as silver [16] or had a mixed composition containing copper [15] and stainless steel filaments [17]. Conductive fibers have also been explored in the integration of surface electrodes into wearable devices’ textiles. In [18], Shafti et al. used stainless steel fiber to embroider surface electrodes for sEMG signal acquisition. The findings indicate that increasing the surface area and fiber density of the electrode contributed to a reduction in the electrical resistance of the electrode. In [19,20], silver-coated conductive fibers with resistances of 60 Ω/m and 530 Ω/m, respectively, were used to knit surface electrodes for electromyographic signal acquisition. In both studies, the results demonstrated that the developed electrodes were capable of effectively acquiring the electromyographic signal.

This paper builds upon the team’s previous work [21], which conceptualized a new wearable for FHP monitoring and correction, and we present as innovative aspects the use of conductive fibers for integrating surface electrodes and conductive tracks into the textile fabric. This design minimizes the need for external cables, thereby increasing the usability and comfort of the device. Further, it combines IMUs and sEMG sensors to provide a more comprehensive ergonomic assessment. This paper also addresses the accuracy and response time of the sEMG acquisition system. The performance evaluation is based on the definition and standardization of requirements, which were the driving guide for this manuscript. Firstly, this study delves into the validation of textile surface electrodes and conductive tracks, including traction and abrasion tests, with various track structures with different thicknesses and orientations being tested. Then, it presents the validation of the developed sEMG acquisition circuit and electrodes by assessing its accuracy and reliability in capturing electromyographic signals and benchmarking it against commercial sEMG sensors, namely, the Trigno Avanti system from Delsys, which is considered a gold standard in terms of sEMG acquisition [22,23]. To achieve this, first, commercial Ag/AgCl electrodes are used to assess and validate the accuracy of the sEMG acquisition circuit by defining its error. Subsequently, the circuit is validated with the produced textile electrodes. Finally, we present the inertial-based posture monitoring modules and the validation of the joint angle estimation algorithm.

The presented manuscript is divided into six main sections. Section 2 outlines the conceptualization of the wearable, with the definition of functionality and usability requirements and the description of the design. Section 3 provides a detailed description of the textile samples used during the validation tests of the acquisition hardware and software. Further, it outlines the validation protocols used for both the textile samples and for the acquisition hardware and software. Section 4 and Section 5 present the results obtained during the validation tests as well as their discussion and contextualization.

## 2. FHP Wearable System Concept and Design

### 2.1. System Requirements

This wearable device was conceptualized to allow real-time and holistic monitoring of upper-body posture: specifically, the FHP. Thus, it encompasses two different modules that provide complementary information, namely, an sEMG module, which provides the electromyographic activity of cervical muscles, and a posture monitoring module, which analyzes the spine’s curvature.

To attain a device that is highly accepted by the end user, it is fundamental to address usability requirements that result in a comfortable and practical device that encourages regular use. Fabric design should consider ventilation and allow heat and sweat dissipation while promoting the unrestricted movement of the user. Integrating the conductive tracks within the textile itself—without the need for bulky electronic units—promotes device integration and aesthetics, which highly influence user acceptance and regular use. Placing the sensors close to the surface of the skin and guaranteeing steady fixation will ensure accurate data collection. Thus, it is necessary to increase fabric compression in the necessary regions. The device should also remain lightweight: ideally under 500 g. Additionally, to increase user engagement and allow easy control of the acquisition modules, the device should offer a user-friendly interface, namely, a smartphone application.

Regarding the device’s electronic requirements, Table 1 presents the main technical requirements considered for the electronics.

One of the core requirements when developing a system is the sampling frequency selection, which should respect the Nyquist theorem, and the frequencies to consider during filter design. In terms of electromyographic signals, the frequencies of the components of interest are typically less than 500 Hz [24]. However, in the 0 to 20 Hz range, there is noise resulting from the firing of the motor units of the central nervous system [25]. Thus, the frequency band of interest should be in the range of 20 Hz to 500 Hz in order to minimize noise and guarantee the acquisition of all pertinent components. Regarding spinal curvature monitoring, the state-of-the-art trend is the use of IMUs. However, these units present some shortcomings: one of the most concerning is the inherent gyroscope measurement drift, which typically results in significant orientation drift. To reduce this effect, strategies such as the strapdown integration (SDI) algorithm can be used, which ideally should be performed with gyroscope data acquired at a high frequency: around 1000 Hz. Data should be available offline, thus demanding a data storage unit, such as an SD card. Additionally, to allow portability and easy system reuse, the system should be powered by a battery and allow integrated recharging.

### 2.2. System Design Proposal

**Figure 1:**
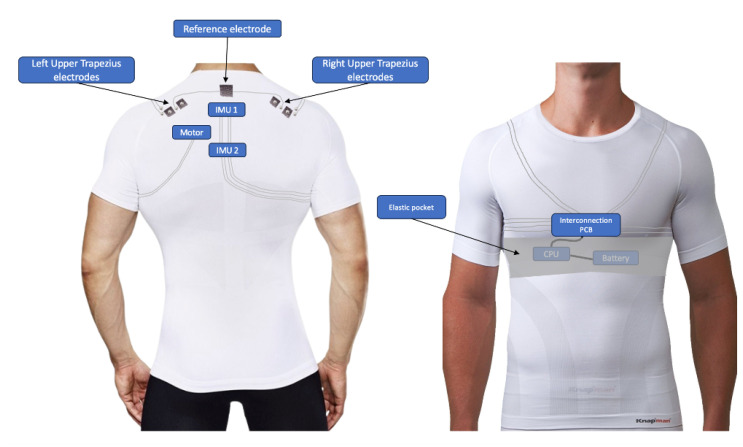
Conceptualized system design.

Figure 1 depicts the design conceptualized for the wearable. FHP predominantly affects the cervical and thoracic portions of the spine. It is characterized by an increase in thoracic kyphosis, which alters the thoracic spine’s curvature [26]. To measure this curvature, the reviewed studies used between two and five IMUs. However, although more sensors can increase accuracy, this also would increase the price and complexity. Therefore, two sensors were placed above the T1 and T5 vertebrae to measure thoracic spine curvature. Additionally, according to the reviewed literature, typically, the upper trapezius is one of the muscles most impacted by FHP [9,10,11]. For this muscle in particular, the Surface Electromyography for the Non-Invasive Assessment of Muscles (SENIAM) project [27] recommends the positioning of the sEMG electrodes at the midpoint between the C7 vertebrae and the acromion, with 20 mm distance between them. To promote aesthetics, an easy and quick setup, usability, and comfort, e-textiles are proposed to embed the electrical connections and sEMG electrodes into the textile substrate.

To maximize the quality and accuracy of the acquired signal, proper fixation of the sensors is essential. To ensure continuous contact between the electrode and the skin, the garment will combine the compression effect, achieved through weft-knitted structures and elastane threads, with a 3D effect obtained from a specific weft knit that significantly increases the thickness of the mesh. These 3D structures are used in areas where textile electrodes are knitted. For example, as presented in [28], a thicker textile knit with a vertical pattern is added in the sensorized regions to ensure greater immobilization of the sensors. The positioning and immobilization of the electrodes at the acquisition locations must be carefully managed, as small discrepancies can generate significant differences in the results obtained. According to [29], positioning the electrodes near the innervation zones can change the acquired signal. For example, in the case of the trapezius muscle, this might result in plateaus appearing in the acquired signal. Additionally, surface electrodes should not be positioned in the most peripheral regions of the muscle to avoid interference from adjacent muscles and the occurrence of cross-talk. Another relevant factor influencing the quality of the acquired signal is the orientation of the electrodes, which should be aligned parallel to the muscle fibers for optimal results [29].

Moreover, to promote healthier postures and posture awareness, a vibrotactile motor is integrated into the design. Snap fasteners are chosen for attaching and removing rigid electronic modules such as printed circuit boards (PCBs), providing a convenient solution for this task. For user comfort, the central processing unit and battery are housed in an elastic pocket on the front of the garment, as illustrated in Figure 1.

## 3. Materials and Methods

The main focus of this manuscript is the validation of the textile component. Specifically, the fiber types, track widths, and electrode dimensions are tested in textile samples of conductive tracks and surface electrodes to verify their electrical properties under mechanical stress and abrasion conditions. Further, we focus on the description of the sEMG signal acquisition modules and their validation as well as the description of the angle estimation algorithms from the IMU data. The validation protocols used for each of the components described are also outlined. Additionally, an Android application developed to control the sEMG acquisition modules using the BLE 4.2 protocol is described.

### 3.1. Textile Electrode and Conductive Track Validation

To test the impact of different structures on the characteristics of conductive tracks and surface electrodes, 22 test samples were designed with varying thicknesses (six and eight courses), orientations (horizontal and vertical), knitted structures (weft-knitted courses with electrically conductive textile yarns combining normal and floating loops), and, in the case of the surface electrodes, surface areas (4 × 2 cm and 2 × 1 cm). The samples were subjected to tensile and abrasion tests to assess the electrical properties’ behavior under mechanical stress and wear conditions.

#### 3.1.1. Test Sample Design

The selection of conductive fibers was made based on criteria such as the electrical characteristics—namely, the electrical resistance—and yarn linear density, since fibers with a high yarn count are thicker, making them more difficult to use during industrial knitting processes. Hence, the selection process prioritized the lowest possible electrical resistance value and a yarn linear density below 300 dtex. Three yarn types were selected, namely, 117/17 HCB [30] and 235/36 HCB [31] from Shieldex (Bremen, Germany) and Elitex Skin Contact [32] from Imbut (Greiz, Germany). The main characteristics of the selected fibers are shown in Table 2.

Looking at the characteristics of the selected fibers, it is possible to observe that the 117/17 HCB fiber has a yarn count of 142 dtex, which is significantly lower than the yarn counts of the 235/36 HCB and Elitex SC fibers, with 295 dtex and 220 dtex, respectively. Therefore, the 117/17 HCB fiber is thinner, making it flexible and easier to use on the mechanical loom. In terms of electrical resistance, the Elitex SC model has the lowest value, with 20 Ω/m, while the Shieldex 117/17 HCB and 235/36 HCB fibers have resistances of 500 Ω/m and 600 Ω/m, respectively.

Given the nature of the knitting process, the orientation of the conductive track on the surface of the base textile can impact its manufacturing process and characteristics. During knitting, the garment is built up row after row, forming interconnecting fiber loops. These loops create horizontal rows (courses) and vertical columns (wales) [33]. The production of a horizontal conductive track is significantly less complex, as it follows the natural orientation of the knitting process, i.e., the textile is naturally knitted in the course’s direction. On the other hand, in a vertical conductive track, knitting involves a constant switch between the conductive fiber and the fiber of the garment’s base textile so that the conductive track follows the alignment of the wales of the knitted structure. Hence, horizontally oriented test samples were designed using both models of Shieldex fibers and the Elitex SC fiber. The vertically oriented test samples were designed using the Elitex SC fiber. Each of the designed conductive tracks has a length of 10 cm.

**Figure 2:**
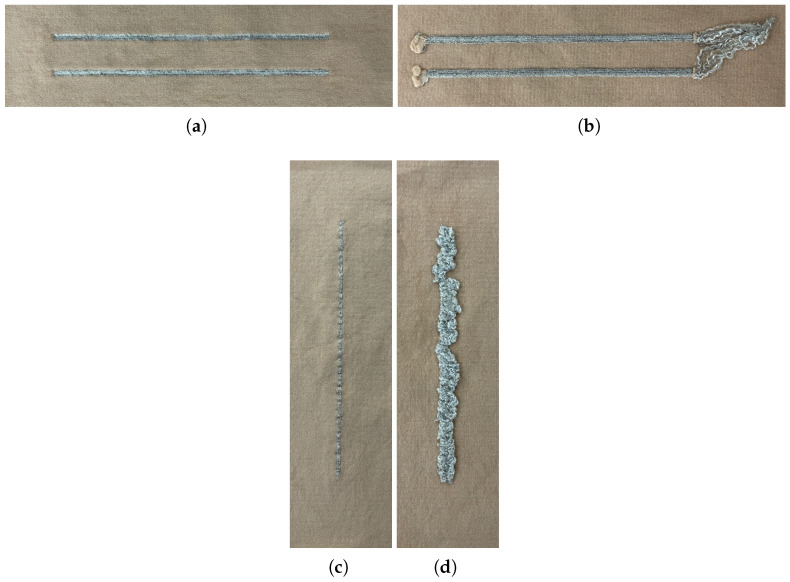
Conductive track test samples (using Elitex SC fiber): (**a**) Horizontal tracks front view. (**b**) Horizontal tracks back view. (**c**) Vertical tracks front view. (**d**) Vertical tracks back view.

Figure 2 presents the designed samples. It can be observed that on the inner side of the vertical tracks (Figure 2d), a surplus of fiber is evident due to the constant switching between the conductive fiber and the base textile fiber. This fiber excess corresponds to the cuts made in the conductive fiber to switch to the base textile fiber. Similarly, to assess the effect of the thickness of the conductive track on its electrical properties, tracks were designed with two different thicknesses of six and eight courses, which translate into widths of approximately 1.9 mm and 2.5 mm, respectively.

**Figure 3:**
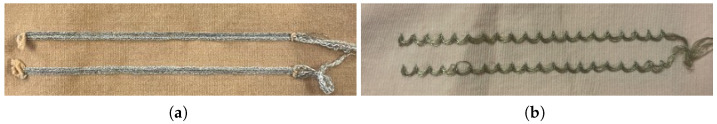
Knitted structures (horizontal tracks): (**a**) Floating loops. (**b**) Base textile courses replaced with conductive fiber.

Besides the thickness and orientation of the conductive tracks, samples with two different knitted structures were also designed, as presented in Figure 3. In one of them, the base textile courses were completely replaced by conductive fibers so that the tracks were fully integrated into the textile fabric of the sample (Figure 3a). The other tested structure consists of the formation of floating loops, meaning that the conductive fiber is knitted only at specific points of the base textile knit, causing loops of conductive fiber to be formed in the interior portion of the sample, connecting the knitted points (Figure 3b). This structure has the advantage of reducing the amount of conductive fiber required, making it possible to reduce costs.

**Figure 4:**
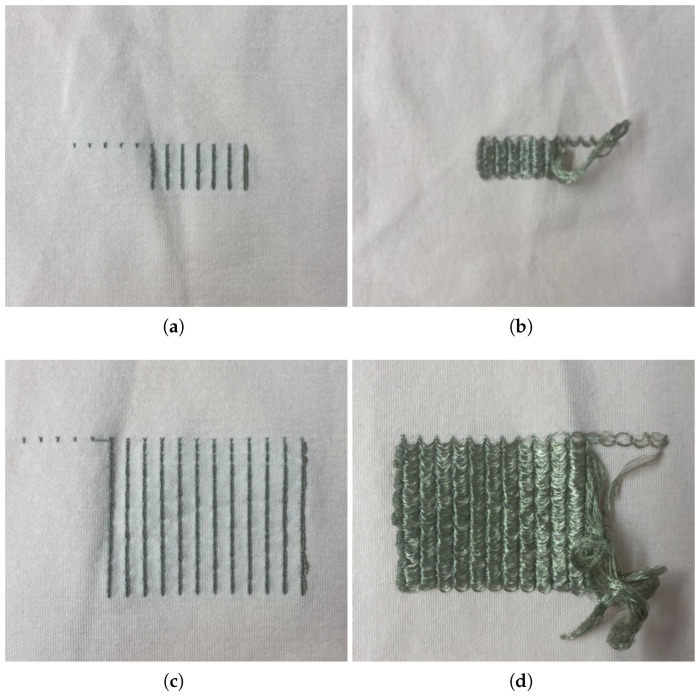
Textile surface electrodes test samples: (**a**) 2 × 1 cm (front view). (**b**) 2 × 1 cm (back view). (**c**) 4 × 2 cm (front view). (**d**) 4 × 2 cm (back view).

Concerning the textile surface electrodes, two designs were tested to evaluate the effect of the surface area on the electrical properties and the ability to acquire the sEMG signal. Both designs have a rectangular shape but differ in surface area (2 × 1 cm vs. 4 × 2 cm). For the electrodes, only the floating loops structure was used, as it enhances contact between the fibers and the skin’s surface. The designed surface textile electrode samples are shown in Figure 4.

#### 3.1.2. Tensile Strength Tests

**Figure 5:**
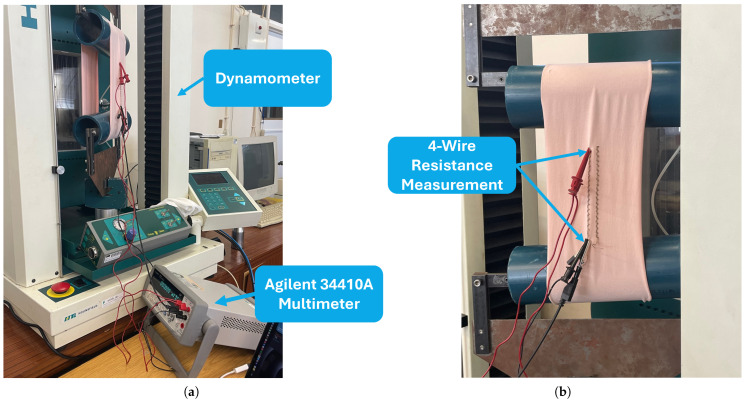
Test setup: (**a**) Dynamometer and digital multimeter setup. (**b**) Four-wire measurement configuration.

Tensile strength tests were conducted on the samples using a dynamometer for which the setup allowed controlled extension of the sample in terms of a maximum value, number of cycles, and constant speed. During the extension tests, the sample resistance was measured using an Agilent 34410A digital multimeter [34] with a four-wire configuration. This configuration ensures accurate resistance measurement by connecting the current and voltage sources to the same point on the sample, mitigating contact resistance between the probes and the sample. The testing setup is illustrated in Figure 5.

To determine the maximum length to which the samples should be stretched, a pre-test was carried out. For the samples with floating loop structures, the maximum value corresponded to the point where the textile loops were fully stretched. In samples where the knitted structure completely replaced the base textile courses, the samples were pulled until they increased their length by 30%.

#### 3.1.3. Abrasion Tests

**Figure 6:**
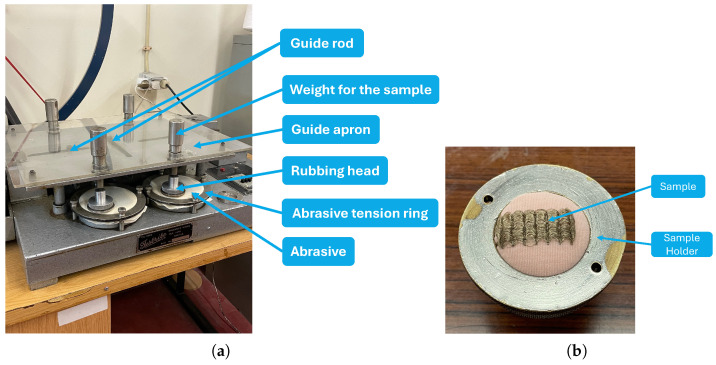
Abrasion test setup: (**a**) Martindale abrasion tester. (**b**) Sample placed inside the rubbing head.

Abrasion tests were carried out to evaluate the durability of the metallic coating on the conductive fibers when exposed to repeated abrasion cycles. The Martindale abrasion tester (Figure 6a) was used for this purpose. The test sample was placed inside the rubbing head (Figure 6b), in contact with the surface of the abrasive, with a weight placed on top of it, allowing the sample to be pressed lightly onto the surface of the abrasive during a cyclic and complex pattern of Lissajous figures [35]. The weight placed on the samples had a mass of 595 g, and the abrasive used was worsted wool.

To measure the electrical resistance of the samples, an Agilent 34410A digital multimeter [34] was used with a four-wire configuration. The resistance was measured for up to 4000 abrasion cycles.

### 3.2. Central Processing Unit

The central processing unit will store and handle data from the IMUs and the sEMG acquisition modules. This unit’s microcontroller is the STM32F405 Feather Express. This microcontroller manages to combine high processing capability with a small size, enhancing usability and user comfort. Table 3 shows the key features of this microcontroller.

Of the features presented, the 168 MHz clock frequency stands out, which is high for a microcontroller with these dimension, allowing it to provide a quicker response to the given inputs, fast data processing, and the execution of more complex data processing algorithms. Other features include an adapter for a microSD card connection and an SDIO interface, which optimizes data writing. The presence of the UART and I2C communication protocols allows communication with the sEMG acquisition modules and the IMUs, respectively. The microcontroller also contains a JST connector and a charging circuit for a LiPo battery, making it easy to power and recharge.

### 3.3. sEMG Acquisition Module

#### 3.3.1. Hardware Development

**Figure 7:**
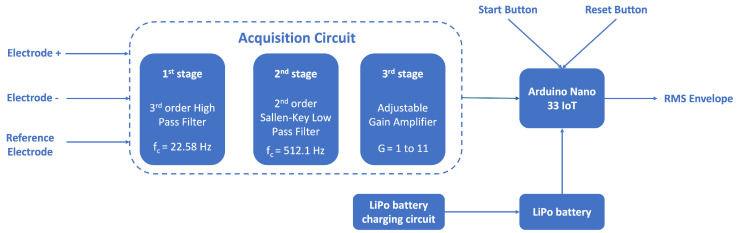
The sEMG acquisition circuit block diagram, with cutoff frequencies of 22.58 Hz and 512.1 Hz.

The sEMG signal acquisition circuit was developed around the AD8232 chip from Analog Devices [36]. This chip allows the sEMG signal to be amplified and filtered simultaneously. It is composed of three stages, as depicted in Figure 7. The first stage corresponds to a third-order high-pass filter that is built around an instrumentation amplifier and is set with a cut-off frequency of 22.58 Hz. The second stage is a low-pass filter with the Sallen–Key typology, which also provides signal amplification. The cutoff frequencies of the filters and the total gain of the signal at the output are defined by dimensioning the passive components connected to the chip. For this system, the Sallen–Key low-pass filter was designed with a cut-off frequency of 512.1 Hz. The gain at the output of the second stage was set to 100. These components were dimensioned using the AD8232 Filter Design software v1.0.2 (Wilmington, MA, USA) [37] from Analog Devices.

To allow additional amplification of the signal, an amplifier with adjustable gain was added and was connected to the output of the AD8232 chip. The gain of the amplifier was set using an AD5241BRZ1M digital potentiometer [38] connected to the feedback loop of the operational amplifier. This potentiometer can be configured to resistance values between 10 KΩ and 1 MΩ, so the gain of this amplifier can vary between 1 and 11.

The sEMG acquisition circuit was interfaced with an Arduino Nano 33 IoT that enables the acquisition of the sEMG signal via the analog-to-digital converter (ADC) and controls the digital potentiometer via I2C. This setup also includes two buttons: one for resetting the Arduino and another for the user to initiate the calibration sequence and start the sEMG signal acquisition.

The Arduino Nano 33 IoT used in the acquisition modules allows for on-site data processing and digital transmission to the central processing unit. This approach reduces the computational load on the STM32F405 Feather Express, which handles data processing and storage. Additionally, it minimizes the risk of data quality loss during transmission to the central processing unit that could arise from using e-textile tracks. Using a wireless body area network (WBAN) is a viable alternative to on-site signal digitalization. However, this approach introduces other challenges such as packet loss and sensor synchronization, which are not the primary focus of this study.

This sEMG module is powered by a LiPo battery. To enable the recharging of the battery, a charging circuit was designed based on the TP4056 chip—to allow a constant current and voltage supply to the battery—and the DW01 chip—to protect the battery from overcharging, undercharging, and overcurrent. The DW01 chip requires two MOSFETs for charging and discharging control, which are present in the FS8205A chip. The charging circuit is connected to the voltage supply via a USB-C connector. To isolate the acquisition circuit from the voltage supply, when charging, a switch was also added.

Synchronization between the Arduino Nano 33 IoT in the sEMG acquisition module and the STM32F405 Feather Express is achieved by sending a flag via UART from the STM32 to the Arduino, which triggers the initiation of sEMG signal acquisition.

**Figure 8:**
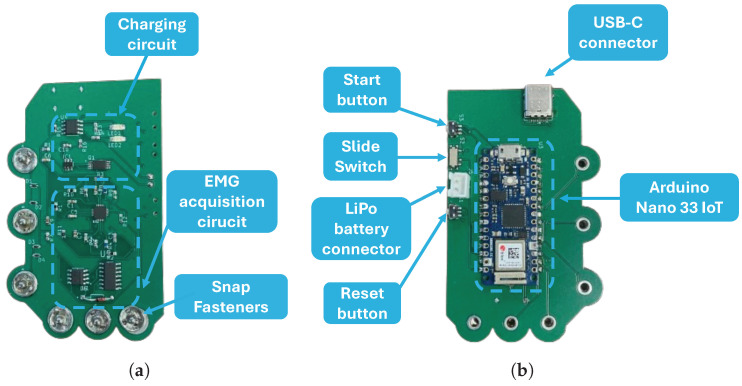
The sEMG acquisition PCB: (**a**) Front view. (**b**) Back view.

The snap fasteners allow the integration and connection of the sEMG electrodes to the developed PCB. Figure 8 illustrates the resulting module, which includes all the aforementioned circuits.

#### 3.3.2. Software Development

The software included in the signal acquisition modules can be divided into four parts: (1) Arduino initialization, (2) calibration sequence, (3) timer interrupt subroutine, and (4) main loop.

During the Arduino initialization process, the I2C and UART peripherals are initialized to configure the digital potentiometer and establish communication with the STM board, and Bluetooth Low-Energy (BLE) is configured to allow communication between the acquisition module and an Android application.

The obtained signals are user-specific, meaning that the average value of one subject is not equivalent to the value of another. Thus, to allow intra- and inter-subject comparisons [39] of the acquired signals, a calibration is conducted whereby the subject’s maximum voluntary contraction (MVC) is acquired over a period of about 3 s, and its root mean square (RMS) value is determined. The MVC value is then used to normalize the signal, which is then expressed as an activation percentage. The timer interrupt subroutine is responsible for signal acquisition and filtering. The analog signal is read at a frequency of 1200 Hz and is digitized based on the Arduino’s reference voltage of 3.3 V and the ADC’s resolution of 12 bits. Then, the signal is filtered, using an infinite impulse response filter (IIR), which is fast and uses fewer coefficients, and, subsequently, less memory, than a finite impulse response filter. Equation (1) presents the generic form of the implemented filter.

(1) $$y\left[n\right]=\left(\sum_{n=1}^{N-1}b_{n}x_{n-N}-\sum_{n=1}^{n=N}a_{n}y_{n-N}\right)\frac{1}{a_{0}},$$

where *a* and *b* represent the filter coefficients, and *N* is the filter’s order. The order chosen for the filter was 12, resulting in 12 coefficients *a* and 12 coefficients *b*, which were determined using Scipy’s *signal* library. The lower and upper cut-off frequencies were set at 20 and 450 Hz, respectively, which are in line with the frequencies used by the Delsys Trigno system [40].

The filtered samples are stored in a buffer. After acquiring and filtering 128 samples, the RMS value is determined, resulting in a new value every 107 ms, or a frequency of 9.34 Hz. After calculating three RMS values, these are sent via UART to the STM board, which translates into a sending frequency of 3.13 Hz.

#### 3.3.3. Android Application

**Figure 9:**
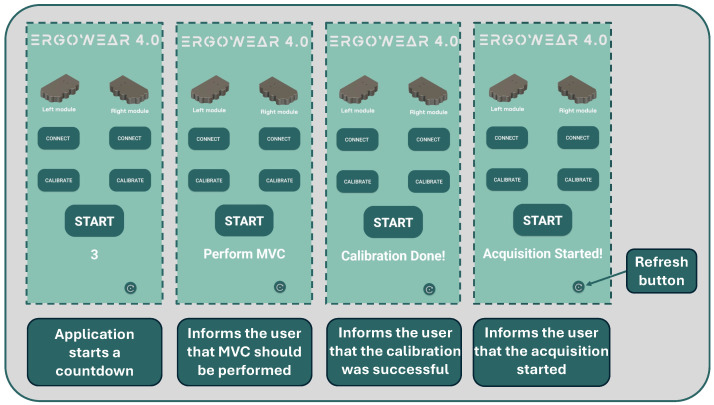
Android application main screens.

**Figure 10:**
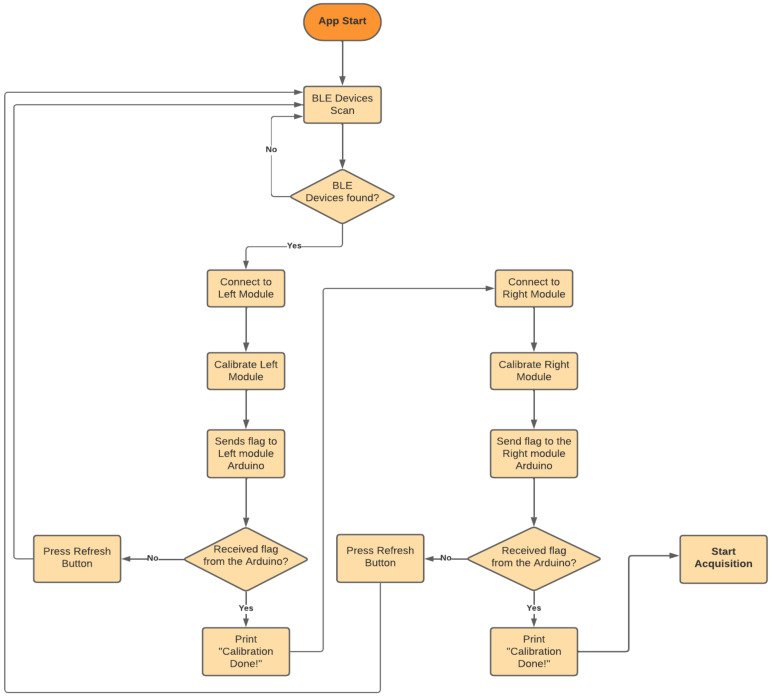
Android application flowchart.

To facilitate control and communication with the signal acquisition modules, an Android application was developed that communicates with the Arduino using the BLE communication protocol. Figure 9 presents the developed application, while Figure 10 depicts the flowchart of the application. The application allows the user to connect to the acquisition modules, start their calibration sequence, and begin signal acquisition. The application starts by searching for available BLE devices in the surrounding area. If the acquisition modules are found, the user can start the connection process with the left module. When the connection is established, the left module can be calibrated by pressing the “Calibrate” button. A countdown starts, and at the end of it, a flag is sent to the Arduino of the acquisition circuit to start the calibration sequence. Once the left module has been calibrated, the user can repeat the process for the right module. After calibration of both modules, signal acquisition can be started by pressing the “Start” button. When this button is pressed, a flag is sent to the Arduino to start acquiring the signal.

#### 3.3.4. Experiments

The quality of the signal acquired by the acquisition circuit was validated against a commercial, gold standard system: the Delsys Trigno Avanti sensor. The sEMG signal was acquired using two types of electrodes with different purposes: (1) commercial Ag/AgCl electrodes to evaluate the acquisition error of the developed sEMG acquisition circuit and (2) the developed textile electrodes to determine the error and assess the quality of the acquired sEMG signal. Data acquisition between systems was synchronized using a hardware trigger system.

#### 3.3.5. Subjects

To validate the electrodes, 11 participants (4 females and 7 males) with ages between 23 and 28 (24.82 ± 1.94) years old and body masses between 50 and 93 (66 ± 12.88) kg voluntarily participated in this study. These participants were selected based on the following inclusion criteria: healthy subjects without clinical history or evidence of motor injuries, namely, shoulder or arm pain. All participants signed a written consent.

#### 3.3.6. Validation Protocol

**Figure 11:**
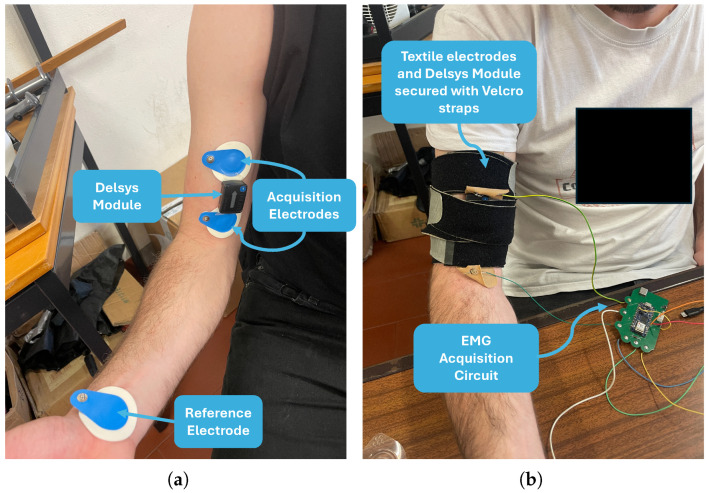
Electrode placement: (**a**) Using Ag/AgCl electrodes. (**b**) Using textile electrodes.

Data acquisition was conducted inside a laboratory in the School of Engineering of the University of Minho under the ethical procedures of the Ethics Committee in Life and Health Sciences (CEICVS 147/2021) and following the standard set by the Declaration of Helsinki and the Oviedo Convention. The validation protocol was the same for both types of electrodes, as depicted in Figure 11. Although the sensory device was designed for the trapezius muscle, the experiments for validating the acquisition hardware and surface electrodes were conducted on the biceps brachii, which is a superficial muscle of the arm. This choice was primarily due to its anatomy, which allowed for easy fixation of the textile electrode samples using a Velcro strap, which is not feasible for the trapezius muscle without the fully developed garment. Additionally, the size of the biceps brachii allowed the simultaneous placement of both the Trigno Avanti sEMG sensors from Delsys and the acquisition module electrodes. The electrodes were positioned according to SENIAM conventions [27], which specify positioning them at one-third of the distance between the acromion and the cubitus fossa. The reference electrode was placed on the subject’s wrist. Figure 11 depicts the electrodes’ positioning. No skin preparation was done in order to replicate the conditions conceptualized for the wearable, where users simply wear the prototype without the need to shave or clean the skin beforehand. The Delsys Trigno system module and the textile electrodes were secured with Velcro straps. All data were synchronized using the Delsys’ hardware trigger. During the calibration process of the acquisition board, the subject performed the MVC for the biceps branchii [27] for a period of 3 s. Two contraction conditions were tested: static, isometric contraction and dynamic contraction consisting of five biceps contractions without any added weight. In static conditions, the subject contracted the biceps until fatigue was self-perceived by the participant.

To allow a comparative analysis, sEMG data from Trigno Avanti sensors were processed offline using the EMGworks Analysis software v4.7.3.0 (Natick, MA, USA) First, data were normalized with the recorded MVC. Then, the signals’ envelopes were calculated using the software’s amplitude analysis, which corresponds to the RMS value calculated with a window of 0.25 s and with no overlap, allowing the signals to be expressed as muscle activation percentages. Data were then exported to a .csv file.

**Figure 12:**
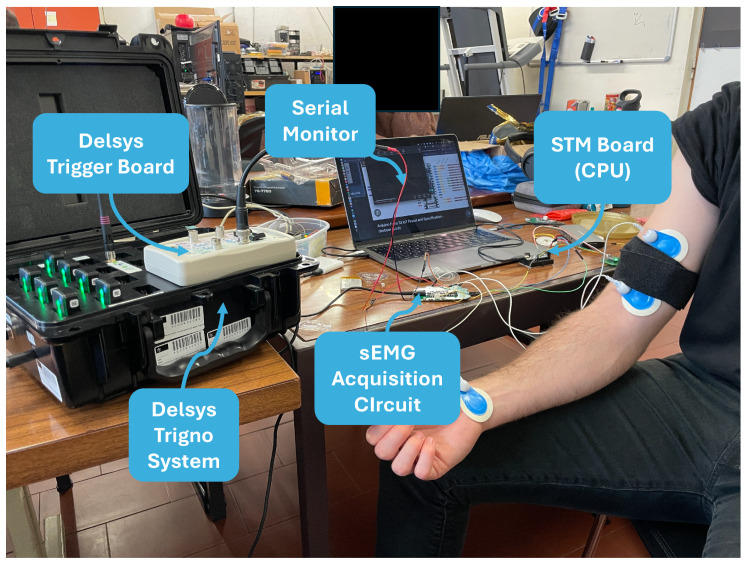
Test setup.

The data from the sEMG acquisition board were displayed on a serial monitor for real-time analysis. Although the sEMG circuit can be powered by a LiPo battery, during the validation experiments, the sEMG circuit was powered via the Arduino Nano 33 IoT, which, in turn, was powered via USB from the computer. The complete test setup is depicted in Figure 12.

### 3.4. Spine Curvature Monitoring Module

#### 3.4.1. Hardware

To estimate the curvature of the thoracic portion of the spine, two IMUS were used and were located at the T1 and T5 vertebrae, which is similar to the setup in [12]. The chosen model was the low-cost LSM6DSOX IMU from Adafruit, which includes a three-axis accelerometer and a three-axis low-bias gyroscope. The main features of this IMU are shown in Table 4.

**Figure 13:**
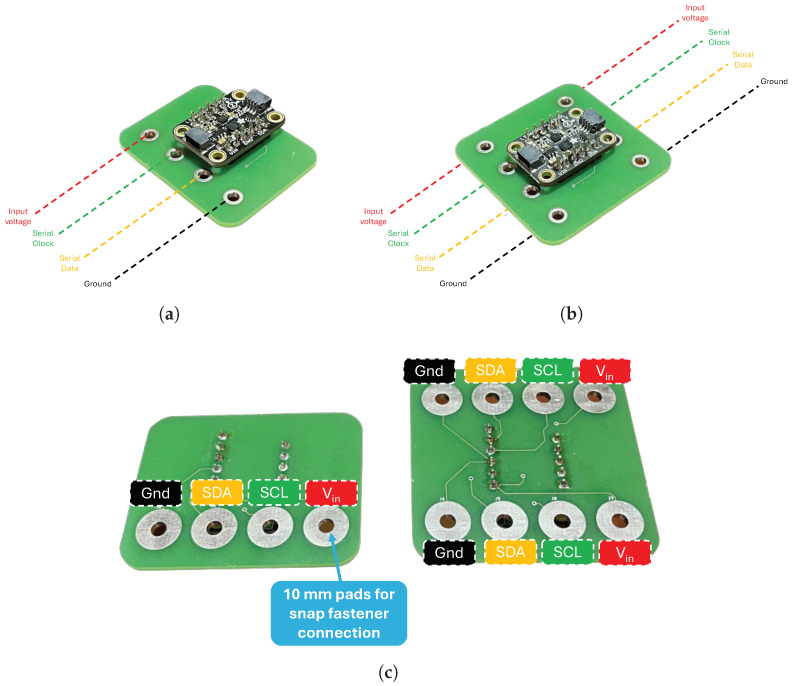
IMUs for PCB design: (**a**) IMU placed above the T1 vertebrae (front view). (**b**) IMU placed above the T5 vertebrae (front view). (**c**) PCBs (back view).

The IMUs are interfaced with the STM board present in the central processing unit via the I2C communication protocol. Since this IMU can assume two possible addresses (0x6A or 0x6B), both IMUs used can be connected to the same I2C bus. To facilitate the connection of the IMUs to the conductive textile tracks, two PCBs were designed with snap fasteners (Figure 13).

#### 3.4.2. IMU Calibration Routine

IMUs are influenced by sensor bias errors. Therefore, a calibration step is required before data acquisition. To this end, raw data are collected for 10 s. In the case of the accelerometer, the average acceleration for each of the axes is determined and used to determine the acceleration norm (Equation (2)), which is then subtracted from the measured acceleration values.

(2) $$Acc_{norm}=\sqrt{Avg\left(Acc_{x}^{2}\right)+Avg\left(Acc_{y}^{2}\right)+Avg\left(Acc_{z}^{2}\right)},$$

In turn, the gyroscope is calibrated by determining an offset, which consists of the average value of the raw angular velocity for each axis. This offset is then subtracted from the following angular velocity values measured by the gyroscope.

#### 3.4.3. Angle Estimation Algorithm

**Figure 14:**
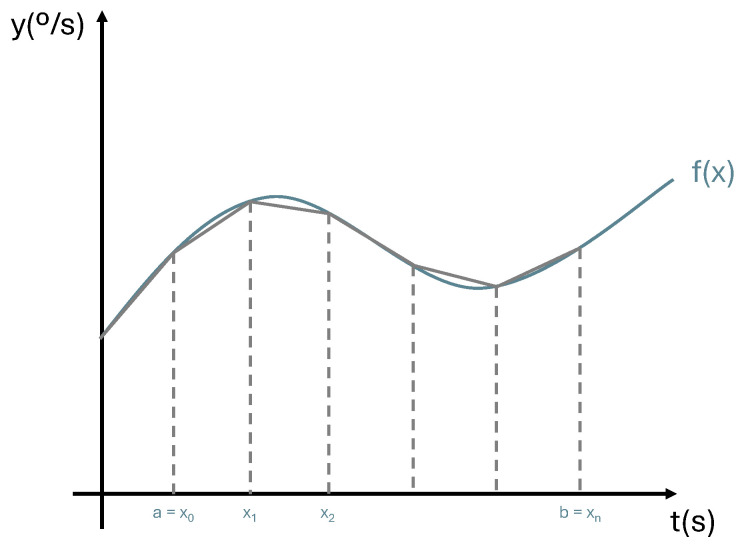
Trapezoidal integration.

The gyroscope’s main problem is related to the accumulation of drift in its angular velocity measurements over time. To reduce this effect, an SDI algorithm is implemented, which consists of acquiring the sensor data at a high frequency and integrating it to determine the sensor’s position or orientation. The SDI algorithm is advantageous due to its high internal sampling frequencies, which allow for more accurate estimations of orientation [41,42]. To determine the gyroscope’s orientation values, a trapezoidal integration method was implemented. This method calculates the area under the curve and approximates it using equally divided trapezoids (Figure 14). This integration acts as a low-pass filter, as it smooths high-frequency components of the signal by averaging successive samples. Specifically, six samples of gyroscope data are collected at a frequency of 100 Hz, and after the sixth sample, the values are integrated. The resulting value corresponds to the orientation of the gyroscope.

To obtain the pitch and roll angle values estimated from the accelerometer acceleration values, Equations (3) and (4) were used, respectively.

(3) $$Pitch\left(\theta \right)=arctan\left(\sqrt{\frac{-Acc_{x}}{Acc_{y}^{2}+Acc_{z}^{2}}}\right),$$

(4) $$Roll\left(\phi \right)=arctan\left(\sqrt{\frac{Acc_{y}}{Acc_{x}^{2}+Acc_{z}^{2}}}\right),$$

where $Acc_{x}$ is the acceleration in the x-axis, $Acc_{y}$ is the acceleration in the y-axis, and $Acc_{z}$ is the acceleration in the z-axis.

To reduce the individual limitations of both accelerometer and gyroscope readings, the final estimated angle value fuses data from both sensors using a linear complementary filter (LCF), for which the implementation is based on [43] and according to Equation (5). The complementary weights, which determine the contributions of the accelerometer and gyroscope data, are given by the α parameter. For the roll and pitch angles, the α parameter assumes values of 0.017794 and 0.018354, as determined empirically.

(5) $$\hat{x}=\alpha \left(\int gyr.dt\right)+\left(1-\alpha \right)acc,$$

#### 3.4.4. Validation Protocol

**Figure 15:**
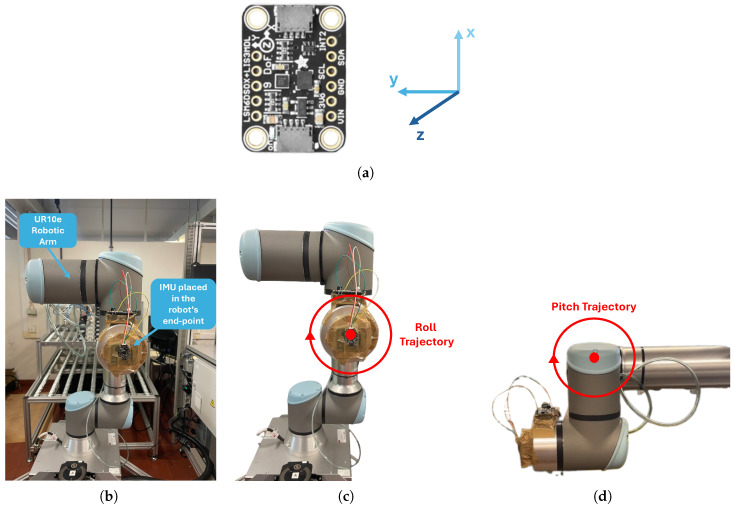
Angle estimation validation: (**a**) IMU reference axis. (**b**) Test setup. (**c**) Roll trajectory. (**d**) Pitch trajectory.

The IMU’s angle estimation algorithm was validated using a UR10e robotic arm from Universal Robots (Odense, Denmark). This is a high-precision robotic arm that is capable of repeating its poses with an accuracy of 0.05 mm [44]. The robot joints can rotate 360 degrees, while its end joint can rotate indefinitely. Data were recorded from the robot’s encoder at a frequency of 500 Hz using ROS 1 middleware and drivers, were saved in a rosbag file, and were then exported to a CSV file. The IMU was fixed at the robot’s end-effector according to the reference in Figure 15a, and the movement around its y- and z-axes was considered as pitch and roll, respectively. Figure 15b depicts the experimental setup.

The trajectory followed by the robot was defined using the PolyScope graphic interface. During the test, movements were performed within a 360° range, with all the robotic joints fixed except the end-joint. The trajectories followed for roll and pitch angle estimation are depicted in Figure 15c,d. Two different speeds were tested: 40°/s, considered a speed comparable to the cervical movement, and an extreme speed of 120°/s, considered only for testing purposes to assess the IMU’s behavior under extreme conditions. For each speed, five 360° rotations were executed in both the pitch and roll directions. The SDI and LCF algorithms were applied to the accelerometer and gyroscope data to estimate the pitch and roll angles, which were then compared with the end-joint angles as measured by the robotic arm.

## 4. Results

### 4.1. Textile Tests

#### 4.1.1. Tensile Strength Tests

**Figure 16:**
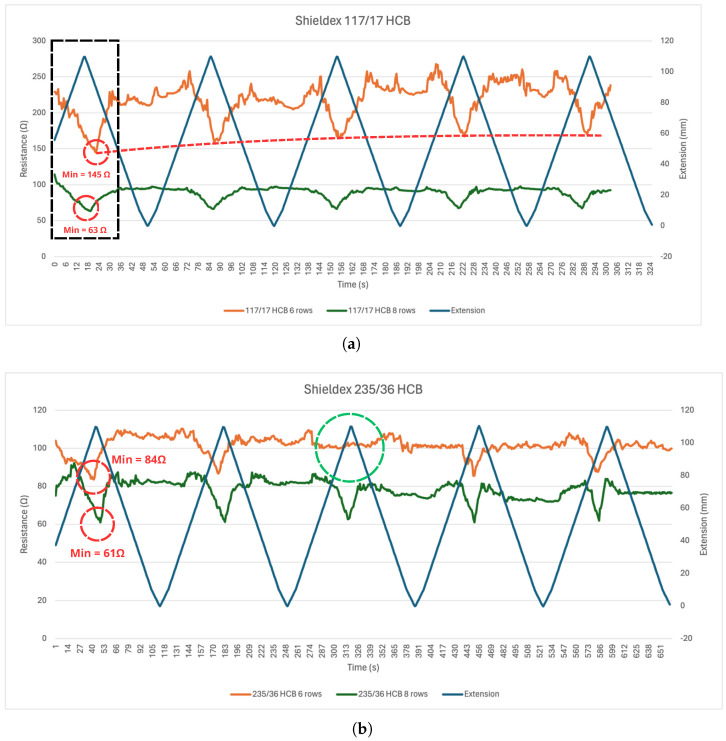
Variation of the resistance of the samples during the tensile strength tests: (**a**) Shieldex 117/17 HCB. (**b**) Shieldex 235/36 HCB. The dashed black square shows the behavior of the fibers during a traction cycle. The red dashed line highlights shows the tendency for the minimum resistance value to increase with each traction cycle. The red dashed circles highlight the maximum and minimum values of resistance. The green dashed circle shows the missing peak due to the acquisition error.

Figure 16 presents the results of the tensile strength tests for samples with Shieldex fibers and a floating loop structure. As can be observed, the resistance of the Shieldex 117/17 HCB sample with six rows of track thickness (Figure 16a) decreased with the extension of the textile (black dashed rectangle in Figure 16), reaching a minimum of 145 Ω at its maximum extension (100 mm). However, the minimum resistance increased during the test, likely due to the fact that the relaxation time between deformation cycles was not enough to allow the fibers to return to their initial shape. Comparably, the samples with 117/17 HCB and eight-row-thick tracks (Figure 16a) reached a minimum resistance of 63 Ω, with a slight increase observed in resistance during each traction cycle due to fabric deformation. The samples with Shieldex 235/36 HCB and eight-row-thick tracks (Figure 16b) had a minimum resistance of 61 Ω at maximum extension. In contrast to the Shieldex 117/17 HCB samples, there was a noticeable decrease in the fluctuation in minimum resistance between traction cycles. This might be explained due to the fiber’s higher tenacity (60 cN/tex for the 235/36 HCB compared to 48 cN/tex for the 117/17 HCB). Regarding the Shieldex 235/36 HCB samples with six-row-thick tracks, the lowest measured resistance was 84 Ω at maximum extension. In contrast to the eight-row sample, there was a more visible change in the minimum resistance between subsequent tensile cycles, suggesting a greater degree of fabric deformation, as seen in Figure 16a as represented by the red dashed line. During the tensile strength test on the six-row sample produced with Shieldex 235/36 HCB fiber, an error in the digital multimeter led to the absence of the variation in electrical resistance values for the third traction cycle, as marked with the green circle in Figure 16b.

**Figure 17:**
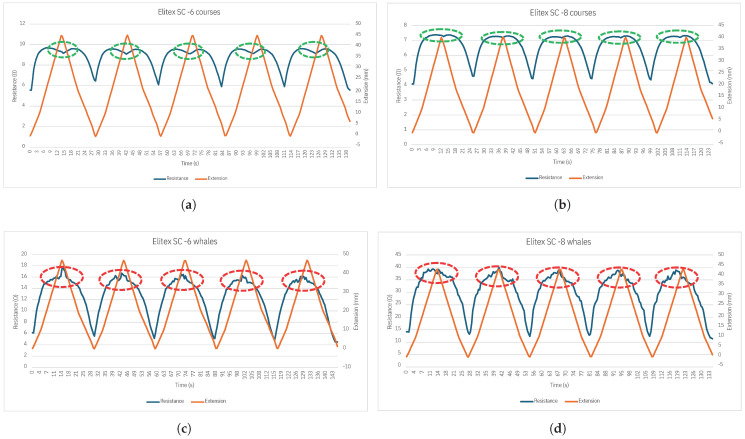
Variation of the resistance of the samples during the tensile strength tests: (**a**) Elitex SC with a thickness of 6 rows (horizontal). (**b**) Elitex with a thickness of 8 rows (horizontal). (**c**) Elitex SC with a thickness of 6 rows (vertical). (**d**) Elitex SC with a thickness of 8 rows (vertical). show the typical piezoresistive behavior of horizontal track samples. The red dashed circles show the more fluctuating behavior of the vertical track samples.

For the samples produced with the Elitex SC fiber and with a knitted structure that replaces the base textile courses with conductive fiber, the achieved results are presented in Figure 17. The tests were conducted until the sample increased its length by 30%, i.e., 40 mm. As traction was applied, the resistance increased, peaking at 9.5 Ω for six-row tracks (Figure 17a) and 7.4 Ω for eight-row tracks (Figure 17b). In the six-row vertical samples, for a stretch close to the maximum stretch tested (between 20% and 30%), it can be observed that the behavior is similar to the typical piezoresistive behavior of these structures, with a decrease in the resistance value as the length of the sample increases, as seen in the green highlighted sections in Figure 17a. For the eight-row sample, however, the variation in the resistance value for a stretch close to the maximum tested extension (between 20% and 30%) is practically null, as depicted in the green highlighted sections in Figure 17b. As for the samples with the vertical track, an increase in electrical resistance was observed as sample length increased. Resistance peaked at 45 Ω for the six-row track (Figure 17c) and 16 Ω for the eight-row track (Figure 17d). For extensions close to the maximum extension tested (between 20% and 30%), the variation in resistance with extension takes on more unstable behavior (highlighted in red in Figure 17c,d), which may be due to the excess fiber inside the sample resulting from the production process of the parallel wefts.

#### 4.1.2. Martindale Abrasion Tests

Table 5 shows the resistance values measured on the different samples during the Martindale abrasion tests.

There were significant changes in all samples with Shieldex fibers. After 1000 abrasion cycles, the resistances in the conductive tracks of both samples produced with Shieldex 117/17 HCB fiber exceeded the multimeter scale value (on the order of GΩ), suggesting substantial damage or possible loss of the metallic coating in some regions, which could have caused a large increase in resistance (highlighted in red in Table 5). For the conductive track samples produced with Shieldex 235/36 HCB fiber, resistance increased, but to a lower degree. For example, resistance in the six-row sample increased from 77 to 333 Ω after the first 1000 cycles and then to 720 Ω after 2000 cycles. However, the resistance exceeded the maximum value measurable by the multimeter after the final trial, showing deterioration of the metallic coating. For the Shieldex 235/36 HCB eight-row sample, the resistance also increased after each test, but the measured values were significantly lower, reaching a peak of 4200 Ω after 3000 cycles. This suggests far less wear of the metallic coating.

The 2 × 1 and the 4 × 2 electrode samples produced with Shieldex 117/17 HCB fiber exhibited significant increases in resistance of 3515% and 17,425%, respectively.

On the other hand, the samples produced with Elitex SC fiber showed some increase in the resistance after subsequent abrasion cycles, but it much less pronounced when compared with the Shieldex’s samples. The Elitex SC samples all remained below 5 Ω. The resistance increased by 207% in the conductive track sample with six rows, 144% in the sample with eight rows (highlighted in green in Table 5), 689% in the 2 × 1 electrode, and 746% in the 4 × 2 electrode. Despite the fact that these increases are noteworthy, it is important to note that the abrasion tests simulate extreme conditions. In contrast, the fibers on a wearable device contact the user’s skin, which has less noticeable abrasive qualities.

### 4.2. sEMG Sensor Validation

Since data from Trigno Avanti sensors and the team’s system were acquired at different sampling frequencies (7.36 Hz vs. 9.34 Hz), it was then processed using MATLAB’s dynamic time warping (DTW) function [45], enabling the alignment of the peak-to-peak envelopes.

**Figure 18:**
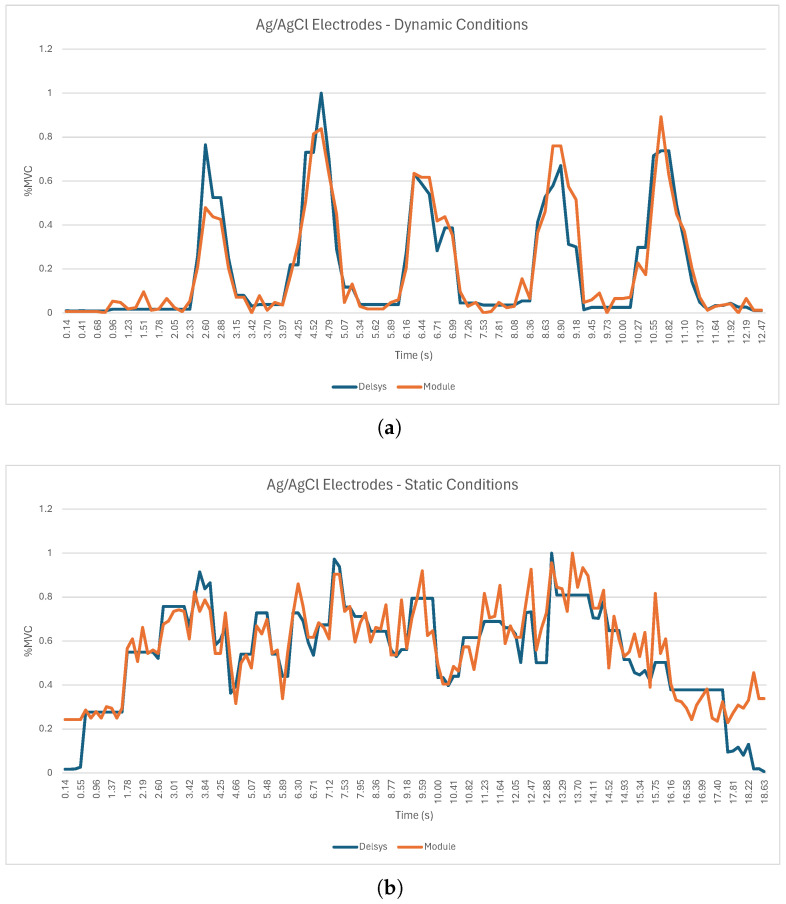
sEMG acquisition using Ag/AgCl electrodes (Subject ID04): (**a**) Dynamic conditions. (**b**) Static conditions.

Figure 18a shows the RMS envelopes during dynamic tests for one participant, showcasing signals recorded by the sEMG acquisition circuit with Ag/AgCl electrodes overlaid with the sEMG reference sensor. Figure 18b shows the RMS envelopes during static tests for the same participant and systems.

**Figure 19:**
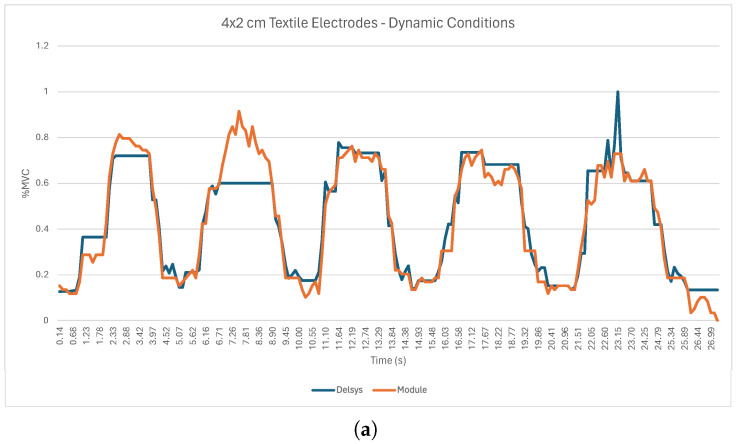

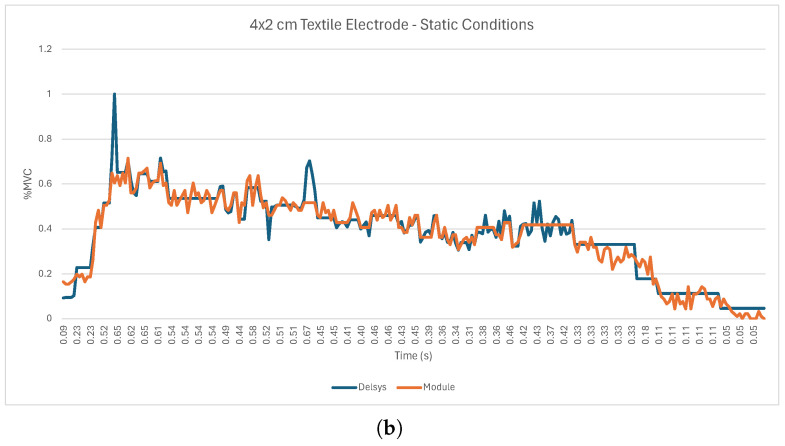
sEMG acquisition using 4 × 2 cm textile electrodes (Subject ID05): (**a**) Dynamic conditions. (**b**) Static conditions.

**Figure 20:**
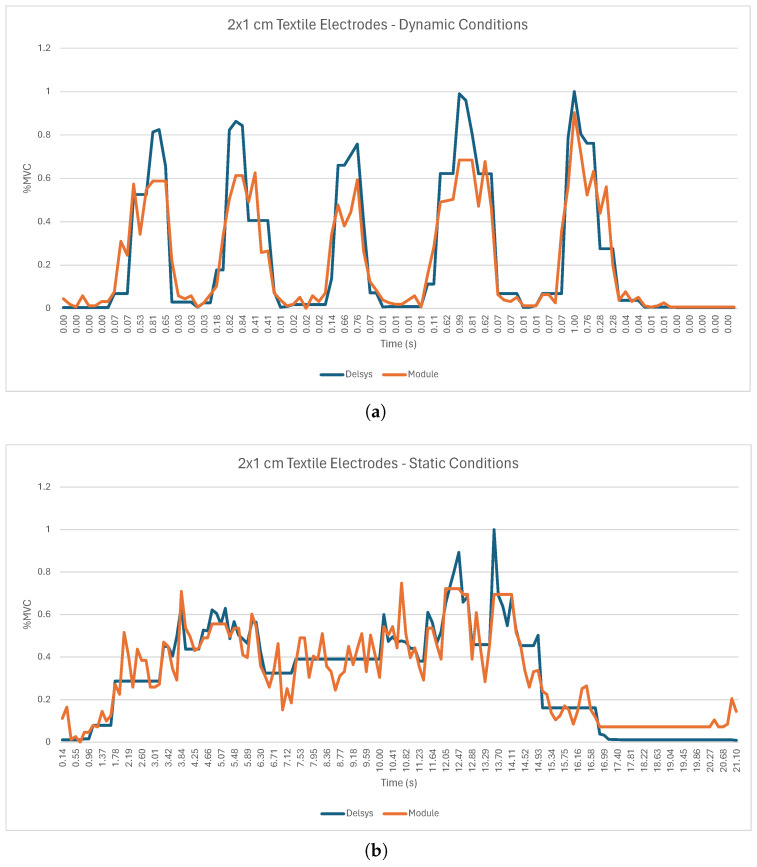
sEMG acquisition using 2 × 1 cm textile electrode (Subject ID011): (**a**) Dynamic conditions. (**b**) Static conditions.

For the dynamic tests with the three types of electrodes (Figure 18a, Figure 19a, and Figure 20a), it is possible to observe five peaks in the sEMG signal, which correspond to the five muscle contractions demanded by the experimental protocol. However, the sEMG signal from the team’s system shows some oscillations, which may result from the fragile connections between the electrodes and the acquisition circuit as well as the presence of electromagnetic and electrostatic interference existing in the area where data acquisition took place. Similarly, for the static test for the three types of electrodes, Figure 18b, Figure 19b, and Figure 20b show that the RMS envelopes of the signals from the acquisition circuit follow the increase in the RMS envelopes of the signals from the Delsys system during the period of isometric contraction of the biceps. However, the signals do contain some oscillations, the origin of which may also be related to the quality of the connections between the electrodes and the acquisition circuit and the presence of noise in the test environment. Table 6 presents the RMSEs computed between the sEMG’s envelopes of the reference sensor, the Trigno Avanti from Delsys, and all the tested electrodes in order to enable a more objective comparison of the systems.

From the analysis of Table 6, starting with the dynamic tests, it is possible to see that the RMSE value for the Ag/AgCl electrodes remained below 14.06% and had an average value of 10.18%. The results for the 4 × 2 cm textile electrodes attained similar values (RMSE < 13.64%, with an average RMSE value of 10.07%, highlighted in green in Table 6). As for the 2 × 1 cm textile electrode, its performance was less consistent, obtaining an average RMSE value of 16.42% and a maximum RMSE of 27.23% (highlighted in red in Table 6). In the static tests, the lowest RMSE value was recorded by the 4 × 2 cm textile electrodes, with an average value of 9.97% (highlighted in green in Table 6). However, the maximum RMSE value recorded by these electrodes was 19.72%. The Ag/AgCl electrodes and the 2 × 1 cm textile electrodes recorded higher average RMSE values, reaching 12.03% and 16.51% (highlighted in red in Table 6), respectively. However, for the case of the Ag/AgCl electrodes, the maximum RMSE value recorded was 17.88%, which explains the lower standard deviation compared to the 4 × 2 cm textile electrodes (3.07% for the Ag/AgCl electrodes in comparison to 5.78% for the 4 × 2 cm textile electrodes).

### 4.3. Angle Estimation Validation

**Figure 21:**
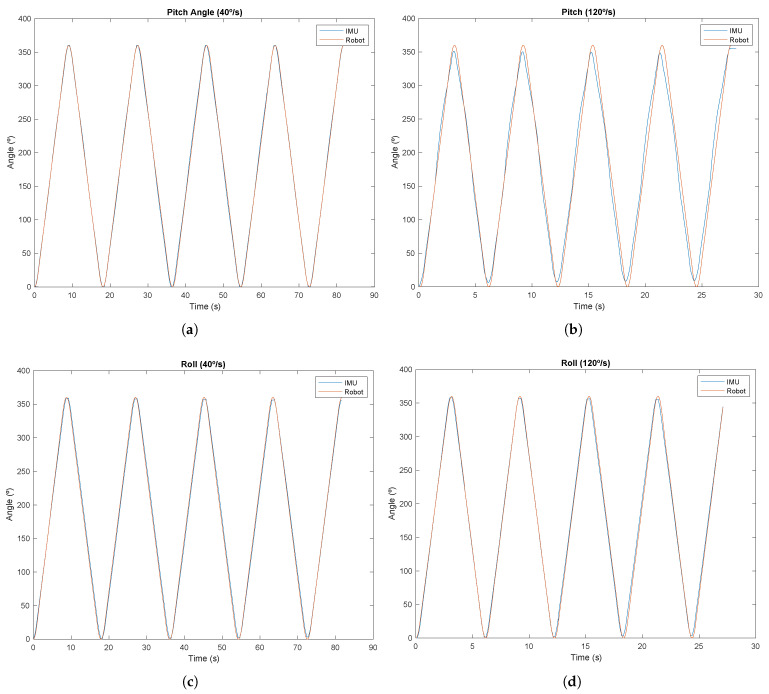
Angle estimation tests: (**a**) Pitch angles at 40°/s. (**b**) Pitch angles at 120°/s. (**c**) Roll angles at 40°/s (**d**) Roll angles at 120°/s.

Table 7 displays the RMSEs for each movement and angular velocity evaluated. The RMSE value for the pitch movement at 40°/s was approximately 3.45°, corresponding to a 0.96% error in relation to the full measured angle range (360°). However, when the angular velocity was increased to 120°/s, the error significantly increased to 10.11°, which corresponds to 2.8% of the movement range. Nevertheless, it is noteworthy that this increased inaccuracy is not concerning, since the angular velocity of forward head movement never reaches such high values. In terms of the roll angles, the RMSE value at 40°/s was 6.09°, which is similar to the RMSE for 120°/s at 6.007°. Figure 21 shows the temporal representation of the angles measured by the team’s system overlaid with the angle data acquired by the UR10e robot’s encoder for each movement (roll/pitch) and angular velocity.

## 5. Discussion

Tensile strength testing on Shieldex samples yielded interesting insights about their mechanical performance. At maximum extension, the resistance of the Shieldex 117/17 HCB samples with a thickness of six rows decreased to 145 Ω, with the minimum resistance value exhibiting discernible variations throughout the traction cycles. The eight-row-thick samples reached a minimum resistance value of 63 Ω, also showing a slight increase in the minimum resistance value throughout the test cycles (Figure 16a). In the samples produced with 235/36 HCB fiber, the minimum values reached were 84 Ω for the six-row-thick sample and 61 Ω for the eight-row-thick sample (Figure 16b). A marginal increase in the minimum resistance value reached at each traction cycle is also measured, but it was to a lesser degree compared with the 117/17 HCB fiber, which can be explained by the higher tenacity of the 235/36 HCB fiber (60 cN/tex compared to 48 cN/tex for the 117/17 HCB fiber), making it more capable of returning to its original form between traction cycles.

In contrast to the Shieldex samples, the Elitex SC fiber samples showed more consistent resistance values during tensile strength tests. With traction, the resistance increased gradually, peaking at 9.5 Ω for the six-row tracks (Figure 17a) and 7.4 Ω for the eight-row tracks (Figure 17b). This suggests that Elitex SC fibers may provide better resistance to mechanical stress compared to Shieldex fibers. In the tests carried out on the samples with horizontal tracks, it can be seen that for traction close to the maximum traction tested (until the initial length increased by 30%), the variation in electrical resistance is significantly lower, which indicates that for the development of a future prototype, it would be appropriate to apply a pre-tension to these structures so that their electrical behavior remains in this range. In the vertical track samples, at extensions close to the maximum extension tested, the behavior is more oscillatory, which may be due to the reorganization of the excess fiber resulting from the production process of these structures. It was also possible to see that for the three types of fiber used, the resistance values (both at rest and during traction) are lower in the eight-row samples than in the six-row samples. In the eight-row-thick samples, there is a higher concentration of knitted conductive fibers, which reduces the electrical resistance and increases the conductivity. Furthermore, analysis of the tensile tests revealed that in structures knitted with floating loops (as seen in Shieldex samples), resistance decreases as tensile strength increases. This decrease in resistance can be explained by the better alignment of the fibers in the loops, which increases conductivity. Conversely, in structures where base textile courses are entirely replaced by conductive fiber (as observed in the Elitex samples), resistance increases with traction.

Martindale abrasion tests offered vital information about the materials’ resilience during long-term wear. Over the course of abrasion cycles, the Shieldex samples showed varied degrees of increases in resistance. Shieldex 235/36 HCB samples showed better resistance to abrasion than Shieldex 117/17 HCB samples. These results are expected due to the higher yarn count and, consequently, greater thickness. Similarly to the results observed in the tensile strength tests, the eight-row-thick samples showed smaller resistance values compared to the six-row-thick samples. However, the samples produced with Elitex SC fibers showed the smallest increases in resistance values, with none of the samples exceeding 4.0 Ω (Table 5). Given the results obtained, a future design will consider using Elitex SC fibers, given their better mechanical and electrical properties, with an eight-row-thick structure for the conductive tracks due to its lower electrical resistance and better resistance to abrasion. With regard to the knitted structure, the substitution of the conductive fiber for the base textile courses generates a more robust structure with less marked variations in resistance when subjected to traction.

The sEMG circuit and electrode validation evaluated the performance of various electrode types under both dynamic and static conditions. The Ag/AgCl electrodes and the 4 × 2 cm textile electrodes revealed similar performance in dynamic testing, with average RMSE values of 10.18 ± 2.83% and 10.07 ± 2.01%, respectively. The average RMSE for the 2 × 1 cm electrodes was significantly higher, reaching 16.42 ± 5.54%. Compared to the 4 × 2 cm electrodes and the Ag/AgCl electrodes, the standard deviation of the 2 × 1 cm electrodes was considerably higher, indicating greater variation between test subjects. In the static tests, the 4 × 2 cm (9.97 ± 5.78%) electrodes showed better results when compared with the Ag/AgCl electrodes (12.03 ± 3.07%) and the 2 × 1 cm textile electrodes (16.51 ± 10.46%) in terms of the average RMSE. However, the standard deviation of the 4 × 2 cm textile electrodes (5.78%) was significantly higher compared with the 4 × 2 cm textile electrodes, suggesting greater instability of results between subjects. These results emphasize the impact that surface area has on the acquisition capacity of textile electrodes, with electrodes with a larger surface area performing better at acquiring sEMG signals under both dynamic and static conditions. This is in agreement with [46], who tested four sizes of textile electrodes (Electrode A: 4 × 3 cm, Electrode B: 1.8 × 3 cm, Electrode C: 4 × 1.8 cm, and Electrode D: 1.8 × 1.8 cm) and demonstrated that a larger area improved signal acquisition in terms of the RMS envelope. Also, according to Barrera et al. [46], a larger surface area is associated with a higher intensity of the acquired signal, which is one of the possible reasons why the performance of 4 × 2 cm electrodes is higher than that of 2 × 1 cm electrodes. Similar results were also verified by [47]. In the future, when developing the prototype and given the results obtained in the textile electrode validation tests, it is considered pertinent to integrate electrodes with the largest possible surface area in order to increase the magnitude of the acquired signal and, therefore, its quality, thus reducing the RMSE as much as possible.

The angle estimation validation demonstrated the accuracy of the IMU-based system for estimating movement angles. For the pitch angle, the smallest error was associated with an angular velocity of 40°/s, with an RMSE of 3.45°. For the 120°/s angular velocity, the RMSE was 10.11°. In the case of the roll angle, the RMSE was similar for both angular velocities, reaching 6.09° for a velocity of 40°/s and 6.007° for a velocity of 120°/s. The results obtained highlight that for movements associated with a higher angular velocity, the error tends to increase, especially for the pitch angle. The higher error value may be due to the accumulation of small errors over time. As the variation in angle amplitude is more abrupt due to a higher angular velocity, there is an accumulation of error resulting from the added noise in the acceleration data from the accelerometer, which can result in an estimated amplitude value that is significantly different from the actual amplitude value. Another factor that could explain the error increase for higher angular velocities is the sensory fusion filter used. Although the complementary filter used is more computationally efficient, for higher angular velocities, for which the raw values from the accelerometer and gyroscope can present additional noise, a more complex algorithm such as a Kalman filter [48] could help mitigate the error in the estimated amplitude. Despite the application of the SDI algorithm, inevitably, there was still residual drift in the gyroscope values, which may also explain the differences between the amplitude measured by the robotic arm and the angle estimated by the IMU. However, for lower angular velocities, as is the case with typical cervical movement, the error remains low (around 0.96% for the pitch angle and 1.69% for the roll angle over a 360° range). These values are comparable to those produced by the state-of-the-art methods [48] (between 2.57° and 4.95° for pitch and between 3.22° and 4.19° for roll) and [49] (RMSE of 3.6° and 5° for the lordosis and kyphosis angles, respectively).

## 6. Conclusions

This work focused on validating the various components required to develop a wearable device for FHP monitoring. Technical and usability requirements were defined and governed the development of the textile and sensory components. In order to evaluate the performance of the conductive fibers when subjected to usage conditions, tensile strength and Martindale abrasion tests were carried out on samples produced with distinct structures and conductive yarns; these tests also made it possible to understand the variations in the electrical properties of the conductive yarns when subjected to these conditions. The development of an electromyographic signal acquisition circuit was also described in detail, along with the algorithms for processing sEMG and inertial data and the Android application for controlling the sEMG acquisition modules.

The tensile test results revealed that samples made with Elitex SC fibers exhibit more stable variations in resistance values. In the samples produced with Shieldex fibers (117/17 HCB and 235/36 HCB), the variations show a more oscillatory behavior. In the abrasion tests, similarly to the tensile strength tests carried out, the samples produced with Elitex SC fibers showed significantly less pronounced variations in resistance values compared to the samples produced with Shieldex fibers, with resistance values remaining below 4.0 Ω after 3000 abrasion cycles. Regarding the acquisition capabilities of textile surface electrodes, it was found that for a larger surface area (4 × 2 cm), the acquired signal is closer to the reference signal from the commercial Delsys system and has a lower RMSE compared to 2 × 1 cm surface electrodes in both static and dynamic conditions. In comparative terms, the performance of the 4 × 2 cm textile electrodes was similar to that of the Ag/AgCl electrodes.

Validation tests of the angle estimation algorithm revealed lower RMSE values for a speed similar to cervical movement (approximately 40°/s), particularly for the pitch angle. In conclusion, the work presented makes it possible to understand how different structures and conductive fibers behave when subjected to mechanical stress and also revealed the capabilities of using e-textiles for sEMG acquisition. These findings will provide useful information for future studies and the design of a functional prototype for FHP monitoring.

Future work will involve the development of a fully integrated prototype that integrates the surface electrodes and conductive tracks into the textile fabric.

## Figures and Tables

**Table 1:** List of electronic requirements.

Definition	Comment
**The system shall be capable of storing data for later analysis**	Data storage on a microSD card
**The system shall be capable of performing strapdown integration**	IMU sampling at 1000 Hz
**The system shall be capable of acquiring signals in the frequency range of interest for sEMG signals**	Frequencies between 20 and 500 Hz
**The system shall be easily rechargeable**	Charging circuit

**Table 2:** Shieldex 117/17 HCB, Shieldex 235/36 HCB, and Imbut Elitex Skin Contact characteristics.

Parameter	Shieldex 117/17 HCB	Shieldex 235/36 HCB	Imbut Elitex Skin Contact
**Electrical Resistivity**	500 Ω/m	600 Ω/m	20 Ω/m
**Yarn count silverized**	142 dtex	295 dtex	235 dtex
**Elongation**	23%	24%	15–25%
**Yield**	70,000 m/kg	33,500 m/kg	22,000 m/kg
**Tenacity**	48 cN/tex	60 cN/tex	31.9 cN/tex

**Table 3:** STM32F405 Feather Express features.

Parameter	STM32F405 Feather Express
**Chip**	STM32F405RGT6
**RAM**	196 kB
**Flash Memory**	1 MB
**Cock Frequency**	168 MHz
**Interfaces**	USB, SPI, I2C, I2S, UART, and SDIO
**Pinout**	14 digital pins 11 PWM pins 6 analog pins
**Dimensions**	23 mm × 52 mm

**Table 4:** LSM6DSOX features.

Parameter	LSM6DSOX
**Input voltage**	1.7–3.6 V
**Current consumption**	0.82 mA
**Accelerometer rate**	6.7 kHz
**Gyroscope rate**	6.7 kHz
**Accelerometer range**	16 g
**Gyroscope range**	±2000°/s
**Dimensions**	26 mm × 18 mm × 5 mm

**Table 5:** Average electrical resistance for Martindale’s abrasion Test. The red lines show the samples most susceptible to degradation during abrasion tests. The green lines show the most resistant samples to abrasion.

Sample	Initial	1000 Cycles	2000 Cycles	3000 Cycles
**Shieldex 235/36 HCB 6 rows**	77 Ω	333 Ω	720 Ω	Break
**Shieldex 235/36 HCB 8 rows**	12 Ω	87 Ω	178 Ω	4200 Ω
**Shieldex 117/17 HCB 6 rows**	78 Ω	**Break**	-	-
**Shieldex 117/17 HCB 8 rows**	86 Ω	**Break**	-	-
**Shieldex Electrode 2 × 1**	1.3 Ω	11.7 Ω	29.4 Ω	47 Ω
**Shieldex Electrode 4 × 2**	**0.97 Ω**	**30.4 Ω**	**60 Ω**	**170 Ω**
**Elitex SC 6 rows**	1.3 Ω	2.1 Ω	3.1 Ω	4.0 Ω
**Elitex SC 8 rows**	0.9 Ω	1.4 Ω	1.8 Ω	2.2 Ω
**Elitex SC Electrode 2 × 1**	0.19 Ω	0.6 Ω	1.1 Ω	1.5 Ω
**Elitex SC Electrode 4 × 2**	0.13 Ω	0.5 Ω	0.8 Ω	1.1 Ω

**Table 6:** sEMG acquisition RMSE. The values highlighted in green showcase the electrodes with better acquisition performance. The values highlighted in red show the electrodes with the worst performance in terms of sEMG acquisition.

Subject	Ag/AgCl (Dynamic)	Ag/AgCl (Static)	4 × 2 cm Electrode (Dynamic)	4 × 2 cm Electrode (Static)	2 × 1 cm Electrode (Dynamic)	2 × 1 cm Electrode (Static)
**ID01**	13.21%	9.19%	8%	7.13%	18.79%	22.12%
**ID02**	11.03%	17.88%	7.7%	9.98%	9.24%	10.14%
**ID03**	6.75%	9.19%	10.68%	9.38%	24.42%	7.83%
**ID04**	7.88%	10.33%	9.17%	11.53%	9.96%	-
**ID05**	14.06%	15.42%	11.22%	4.76%	14.68%	13.44%
**ID06**	13.74%	15.11%	13.64%	19.72%	16.45%	23.47%
**ID07**	10.85%	14.09%	9.74%	7.2%	27.23%	35.31%
**ID08**	11.85%	9.18%	7.46%	8.44%	14.33%	30.02%
**ID09**	7.76%	11.24%	9.19%	12.28%	15.9%	12.9%
**ID010**	6.6%	9.27%	11.71%	-	17.45%	17.93%
**ID011**	8.29%	11.39%	12.27%	19.25%	12.17%	8.45%
**Average ± Standard Deviation**	(10.18 ± 2.83)	(12.03 ± 3.07)	**(10.07 ± 2.01)**	**(9.97 ± 5.78)**	**(16.42 ± 5.54)**	**(16.51 ± 10.46)**

**Table 7:** RMSE of the angle estimation for each direction and speed.

	40°/s	120°/s
**Pitch**	3.45°	10.11°
**Roll**	6.09°	6.007°

## Data Availability

The raw data supporting the conclusions of this article will be made available by the authors on request.

## Abbreviations

The following abbreviations are used in this manuscript:

FHP	forward head posture
IMUs	inertial measurement units
sEMG	surface electromyography
EMG	electromyography
RMSE	root mean square error
SDI	strapdown integration
SENIAM	Surface Electromyography for the Non-Invasive Assessment of Muscles
PCB	printed circuit board
ADC	analog-to-digital converter
WBAN	wireless body area network
BLE	Bluetooth Low-Energy
MVC	maximum voluntary contraction
RMS	root mean square
LCF	linear complementary filter
DTW	dynamic time warping
